# Prevalence of autoantibody responses in acute coronavirus disease 2019 (COVID-19)

**DOI:** 10.1016/j.jtauto.2020.100073

**Published:** 2020-11-27

**Authors:** L. Angelica Lerma, Anu Chaudhary, Andrew Bryan, Chihiro Morishima, Mark H. Wener, Susan L. Fink

**Affiliations:** Department of Laboratory Medicine and Pathology, University of Washington, Seattle, WA, USA

**Keywords:** SARS-CoV-2, COVID-19, Autoantibody, Antinuclear antibody, Antiphospholipid antibody

## Abstract

Immunopathology may play a significant role in the pathogenesis of Coronavirus-Induced Disease-19 (COVID-19). Immune-mediated tissue damage could result from development of rapid autoimmune responses, characterized by production of self-reactive autoantibodies. In this study, we tested specimens from acutely ill patients hospitalized with COVID-19 for autoantibodies against nuclear, vasculitis-associated, and phospholipid antigens. Detectable autoantibodies were present in 30% of the patients in our cohort, with the majority of reactive specimens demonstrating antibodies to nuclear antigens. However, antinuclear antibodies were only weakly reactive and directed to single antigens, as is often seen during acute infection. We identified strongly reactive antibodies to nuclear antigens only in patients with a prior history of autoimmune disease. In our cohort, the prevalence of antiphospholipid antibodies was low, and we did not detect any vasculitis-associated autoantibodies. We found similar levels of inflammatory markers and total immunoglobulin levels in autoantibody positive versus negative patients, but anti-SARS-CoV-2 antibody levels were increased in autoantibody positive patients. Together, our results suggest that acute COVID-19 is not associated with a high prevalence of clinically significant autoantibody responses of the type usually associated with autoimmune rheumatic disease.

## Highlights

• Autoantibodies against nuclear antigens are detectable in 25% of patients hospitalized with acute COVID-19.
• Anti-nuclear antigen antibodies were weakly reactive and most often directed to single antigens.
• Vasculitis-associated autoantibodies were not detected in specimens from patients with acute COVID-19.
• Anti-phospholipid antibodies were infrequently detected in patients with acute COVID-19.

## 1 Introduction

Severe acute respiratory syndrome coronavirus-2 (SARS-CoV-2) is a recently emergent, now pandemic virus and etiological agent of Coronavirus Induced Disease-19 (COVID-19) [1,2]. SARS-CoV-2 infection is characterized by a wide range of clinical outcomes, from asymptomatic infection to severe lower respiratory tract damage and acute respiratory distress syndrome (ARDS) [3]. Lethal disease is often associated with sepsis, coagulopathy, multi-organ failure and heightened inflammatory responses, including cytokine storm syndrome [4]. In addition, a myriad of other clinical associations have been described, including autoimmune phenomena such as Kawasaki-like syndrome, multisystem inflammatory syndrome, Guillain Barre syndrome, immune thrombocytopenic purpura and chilblain-like lesions [5,6].

Immune mechanisms likely play a significant component to the pathogenesis of disease in COVID-19. SARS-CoV-2 infection may trigger development of autoimmunity in susceptible patients, potentially as a result of viral cross-reactivity with autoantigens [7,8]. Nuclear antigens are commonly targeted by autoantibodies, and antinuclear antibodies are a hallmark of multiple autoimmune diseases, including systemic lupus erythematosus [9]. Reactivity to more than one nuclear antigen is characteristically found in specimens from patients with autoimmune diseases. Recent data from small studies suggest a high prevalence of antibodies against nuclear antigens in severe COVID-19, detectable in 92% of 11 ICU patients in a study from Germany [10] and 50% of 21 ICU patients in a study from China [11].

In addition to nuclear antigens, many other self-antigens can be targeted by autoantibodies in association with diverse autoimmune phenomena, including vasculitis and thrombosis. Histologic evidence of vasculitis has been described in COVID-19, suggesting the possibility that autoimmune-mediated vessel diseases may occur [12,13]. In addition, coagulopathy and thrombosis is a prominent feature of severe COVID-19 [14]. Case reports suggest that antiphospholipid antibodies can mediate autoimmune thrombosis in this disease [15].

We sought to better understand the prevalence of autoantibody responses in acute COVID-19, and measured antibodies against nuclear, vasculitis-associated, and phospholipid antigens in specimens from patients hospitalized at our institution.

## 2 Methods

### 2.1 Study design and participants

Remnant serum and plasma specimens from 64 patients with RT-PCR confirmed SARS-CoV-2 infection were collected from the clinical laboratories at the University of Washington and Harborview Medical Centers in Seattle, Washington between March and May of 2020. Specimens were collected under an IRB-approved waiver of consent and stored at −80 ​°C before autoantibody testing. Samples from healthy blood donors in our region obtained prior to the COVID-19 pandemic were used as normal controls.

Medical records were reviewed for history of autoimmune diseases and lab values. When available, the white blood cell count (WBC), C-reactive protein (CRP), ferritin, fibrinogen, and interleukin-6 (IL-6) values were recorded for the date closest to specimen collection within a two-week window in the same hospitalization.

### 2.2 Anti-nuclear antibody detection

Antibodies to nuclear antigens were detected using the BioPlex 2200 antinuclear antibody (ANA) screen multiplex autoimmune assay (Bio-Rad Laboratories). This system measures IgG autoantibodies to the following antigens: double-stranded DNA (dsDNA), centromere B, chromatin, ribosomal protein, SS-A (including both Ro52 and Ro60), SS-B, Sm, Sm/RNP, ribonucleoprotein (RNP), Scl-70/topoisomerase I, and Jo-1. Quantitative results were expressed as an antibody index (AI) for all antigens except dsDNA, which is reported in IU/mL. For our initial analysis, we used the manufacturer’s suggested thresholds, which define <1.0 AI as negative and ≥1.0 AI as positive for antibodies to centromere B, chromatin, ribosomal protein, SS-A, SS-B, Sm, Sm/RNP, RNP, Scl-70, and Jo-1. The manufacturer’s suggested ranges for antibodies to dsDNA are ≤4 IU/mL (negative); 5–9 IU/mL (indeterminate) and ≥10 IU/mL (positive).

Our clinical laboratory has established modified cutoffs for result reporting, based on testing specimens from 264 healthy regional blood donors. Samples with values ​< ​0.8 AI are reported as negative for antibodies to centromere B, chromatin, ribosomal protein, SS-A, SS-B, Sm, Sm/RNP, RNP, Scl-70, and Jo-1. We have established an indeterminate range of 0.8–5.0 AI for centromere B, chromatin, ribosomal protein, Sm/RNP, RNP, and Scl-70. Indeterminate ranges for other antigens are: SS-A and Jo-1 (0.8–1.2 AI), SS-B (0.8–6.0 AI) and Sm (0.8–3.0 AI). Values above the indeterminate range are reported as positive. For anti-dsDNA antibodies, our negative range is ​< ​4 IU/mL; indeterminate is 4–120 IU/mL and positive is ​≥ ​120 IU/mL.

Specimens demonstrating reactivity for any nuclear antigen were additionally tested using indirect immunofluorescence on HEp-2 ​cells (Bio-Rad Laboratories) at a dilution of 1:40.

### 2.3 Detection of vasculitis-associated and anti-phospholipid antibodies

IgG autoantibodies to the vasculitis-associated antigens myeloperoxidase, proteinase 3, and glomerular basement membrane were detected using the BioPlex 2200 Vasculitis panel (Bio-Rad Laboratories). Antiphospholipid syndrome (APLS)-associated antibodies were measured using the BioPlex 2200 APLS multiplex platform (Bio-Rad Laboratories) to detect IgG and IgM anti-cardiolipin and anti-beta-2 glycoprotein I antibodies. Testing for anti-phosphatidylserine/prothrombin complex antibodies was performed using QUANTA Lite aPS/PT IgG and IgM ELISA kits (Inova Diagnostics). All testing was performed according to the manufacturer’s instructions, including verification of calibrators and controls. Quantitative results were classified as negative or positive according to the method’s suggested numerical cutoff.

### 2.4 Quantification of total and anti-SARS-CoV-2 antibodies

Total IgG, IgG subclasses and IgM were measured using the Optilite turbidimetric analyzer (The Binding Site). Testing for anti-SARS-CoV-2 spike protein (S1 domain) IgG antibodies was performed using a semiquantitative immunoassay from EUROIMMUN. The ratio of optical density for each patient sample over calibrator was calculated, and the recommended threshold for positivity was used for interpretation. All assays were performed according to the manufacturer’s instructions.

### 2.5 Statistical analysis

Comparisons between groups was performed using Fisher’s exact test for categorical variables and Mann–Whitney U test for continuous variables.

## 3 Results

### 3.1 Study group characteristics

Our cohort consisted of 64 patients with an RT-PCR confirmed diagnosis of COVID-19, including 27 (41%) receiving care in the intensive care unit (ICU) at the time of sample collection. Specimens were obtained an average of 12.3 days after diagnosis (range: 4–37 days). The mean age of the subjects was 58.3 (range: 19–97 years). The majority of patients recovered, but there were 14 deaths (21%). After specimen collection and testing, retrospective chart review indicated that seven patients had a prior history of autoimmune disorders, including two patients with systemic lupus erythematosus and one patient each with psoriatic arthritis, Crohn’s disease, granulomatosis with polyangiitis, Grave’s disease and multiple sclerosis.

### 3.2 Anti-nuclear antibodies in acute COVID-19

We used a well-characterized, clinically-validated multiplex immunoassay to detect autoimmune rheumatic disease-related autoantibodies to nuclear antigens. The sensitivity of this assay for anti-nuclear antibodies in systemic autoimmune disease is similar to that of other methods [[16], [17], [18], [19]].

**Fig. 1:**
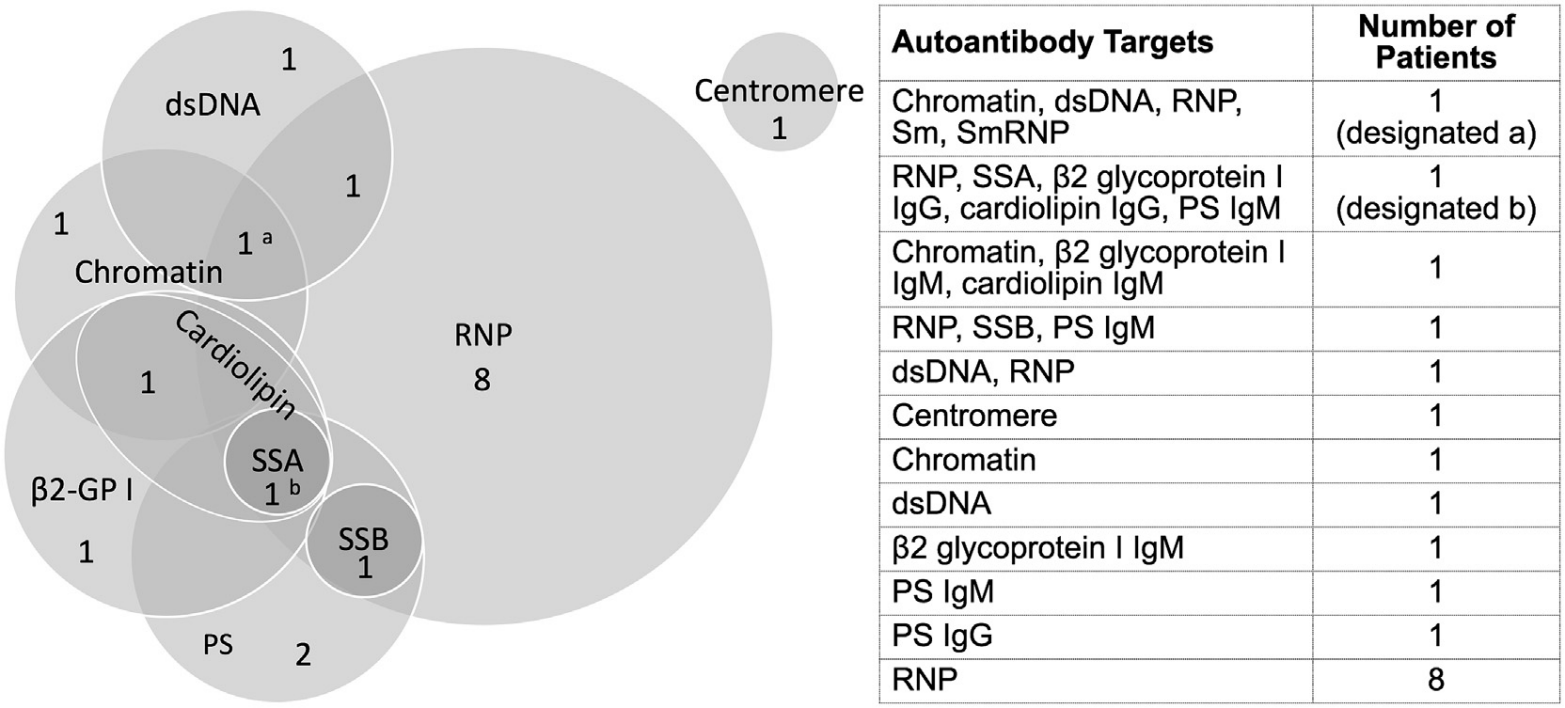
**Antigens Targeted by Autoantibodies in COVID-19 Patients.** The Venn diagram and table illustrate the number of patients with autoantibody combinations against nuclear and phospholipid antigens. Two patients had a history of systemic lupus erythematosus (denoted by superscript letters a and b). Patient a was also positive for Sm and SmRNP.

We detected anti-nuclear antibodies in 25% (16/64) of COVID-19 patients using manufacturer-recommended thresholds. Of the reactive specimens, 75% (12/16) were from patients with severe disease who received care in the ICU. The majority of positive specimens demonstrated reactivity to single nuclear antigens (Fig. 1), with RNP most common (n ​= ​8). Other nuclear antigens targeted were chromatin, centromere B, SS-A, SS-B and dsDNA. No antibodies against ribosomal protein, Scl-70/topoisomerase I, or Jo-1 were detected. Two patients (designated as “a" and “b" in Fig. 1) with reactivity to multiple antigens had a history of systemic lupus erythematosus and positive antinuclear antibody testing prior to SARS-CoV-2 infection. Patient “a” had prior reactivity against dsDNA, chromatin, RNP, Sm, and SmRNP, which was an identical pattern found on retesting during acute COVID-19. Patient “b” had a history of reactivity to SS-A and phospholipid antigens, but reactivity to RNP was not previously demonstrated. All 16 specimens demonstrating reactivity for any nuclear antigen were additionally tested using indirect immunofluorescence on HEp-2 ​cells at 1:40 serum dilution and demonstrated at least trace reactivity, the majority in a speckled pattern.

Although the quantitative results for these autoantibody assays exceeded the manufacturer’s suggested threshold for classification of a positive result, our clinical laboratory uses modified thresholds for diagnostic reporting, based on evaluation of our local healthy control population. Our previous reference range studies using specimens from healthy regional blood donors obtained prior to the COVID-19 pandemic demonstrated a 12% positivity rate using the manufacturer’s cutoffs; all but one specimen was positive for only a single antibody. Indeterminate anti-dsDNA results constituted an additional 4% of the samples. The positive healthy control samples produced quantitative results that were marginally above the manufacturer’s suggested threshold. Based on these results, we established laboratory-defined cutoffs for result interpretation to include or expand an indeterminate range for reporting anti-nuclear antibodies. Using our laboratory-defined thresholds, only two of the COVID-19 patients would have been reported as positive for anti-nuclear antibodies, and these were the two with a history of systemic lupus erythematosus. The remaining 14 would have been reported as indeterminate.

### 3.3 Autoimmune vasculitis-associated antibodies in acute COVID-19

As COVID-19 is associated with pulmonary and vascular manifestations, we also investigated autoantibodies targeting myeloperoxidase (MPO), proteinase 3 (PR3) and glomerular basement membrane (GBM). These antibodies are a hallmark of anti-neutrophil cytoplasmic antibody (ANCA)-associated vasculitides (anti-MPO and PR3) and Goodpasture’s syndrome (anti-GBM). None of the specimens from patients with COVID-19 demonstrated reactivity with any of these antigens.

### 3.4 Anti-phospholipid antibodies in acute COVID-19

The coagulopathy seen in patients with COVID-19 raises concerns that anti-phospholipid antibodies may play a contributing factor [15]. We tested specimens for antibodies against cardiolipin and beta-2 glycoprotein I, which are components of the classification criteria for anti-phospholipid syndrome [20]. Specimens from three patients contained detectable anti-phospholipid antibodies (Fig. 1). One patient had strong IgG reactivity to beta-2 glycoprotein I and cardiolipin as well as antinuclear antibodies. This was the previously described patient “b" who had a prior history of anti-phospholipid antibodies and diagnosis of systemic lupus erythematosus. Another patient was positive for IgM antibodies to both beta-2 glycoprotein I and cardiolipin, and also had anti-chromatin antibodies. The third had only IgM antibodies to beta-2 glycoprotein I.

We then determined the presence of anti-phosphatidylserine/prothrombin complex antibodies, which have also been described in antiphospholipid syndrome [21]. Three patients tested positive for anti-phosphatidylserine/prothrombin complex IgM antibodies and one for IgG antibodies. Two of these patients also had other antinuclear and/or anti-phospholipid antibodies (Fig. 1). Together, we detected anti-phospholipid antibodies in six of 64 (9%) patients with COVID-19. None of these patients had clinical suspicion of COVID-19-related thromboembolism during the acute hospitalization by medical record review.

### 3.5 Correlation of autoantibodies with clinical features, inflammatory markers and immunoglobulin levels

Overall, we detected autoantibodies in 19 (30%) of the COVID-19 patients in our cohort using manufacturer’s thresholds (Table 1). We did not find a statistically significant difference in the age of patients who were positive or negative for autoantibodies. The time interval between SARS-CoV-2 PCR diagnosis and specimen acquisition did not differ between specimens with or without autoantibodies. Patients with a detectable autoantibody were more likely to be in the ICU than those who had no immunoreactivity (63.2% vs 31.1%, *p* ​= ​0.03). However, deaths from COVID-19 did not demonstrate statistical significance between both groups (26.3% vs 20%, *p* ​= ​0.74). Compared to patients with no detected autoantibodies, the rate of pre-existing autoimmune disorders was higher in those patients with positive screens (26.3% vs 4.4%, *p* ​= ​0.02). In patients without known pre-existing history of immunologically-mediated disorders, autoantibodies were found in 26.3% (15/57).

**Table 1:** Characteristics of Patients who were negative for all autoantibodies assessed in this study versus positive for at least one autoantibody, using the manufacturer’s suggested thresholds for each assay.

	Autoantibody Negative (N ​= ​45)	Autoantibody Positive (N ​= ​19)	p Value
Median Age (Range)	55 (19–97)	57 (27–89)	0.58
Days after positive SARS-CoV-2 PCR	10 (4–37)	11 (4–28)	0.25
Patients with prior autoimmune diagnoses	2 (4.4%)	5 (26.3%)	0.02
ICU	14 (31.1%)	12 (63.2%)	0.03
Deaths from COVID	9 (20%)	5 (26.3%)	0.74

**Fig. 2:**
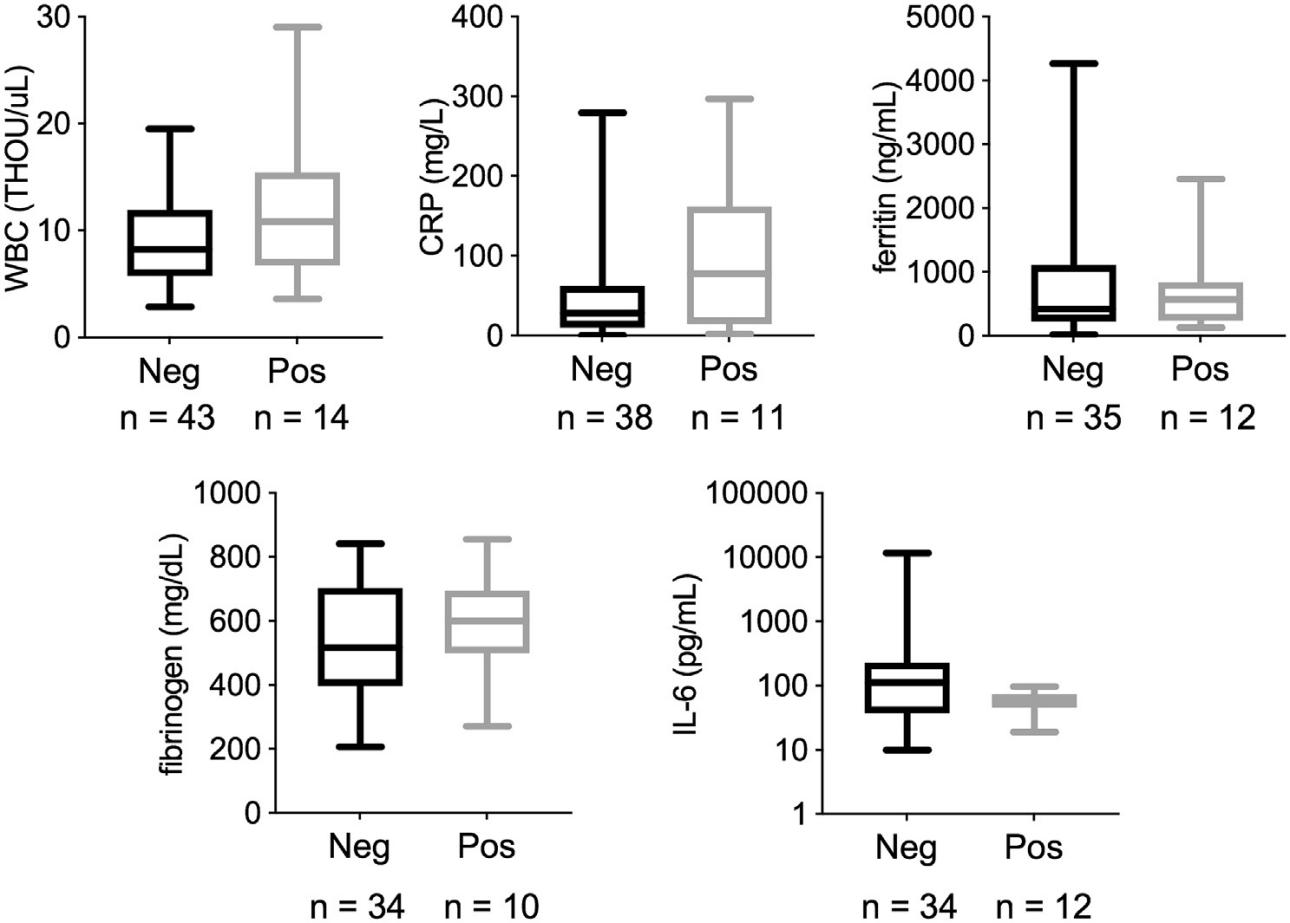
**The Presence of Autoantibodies is Not Associated with Markers of Inflammation.** Laboratory values for patients without history of autoimmune disorders who were negative for all autoantibodies assessed in this study (Neg) versus positive for at least one autoantibody (Pos). Autoantibodies were classified using the manufacturer’s suggested thresholds for each assay. Box-and-whisker plots show median and 25th to 75th percentiles; whiskers indicate minimum and maximum values. No laboratory values were found to be statistically different between groups using the Mann–Whitney U test.

**Fig. 3:**
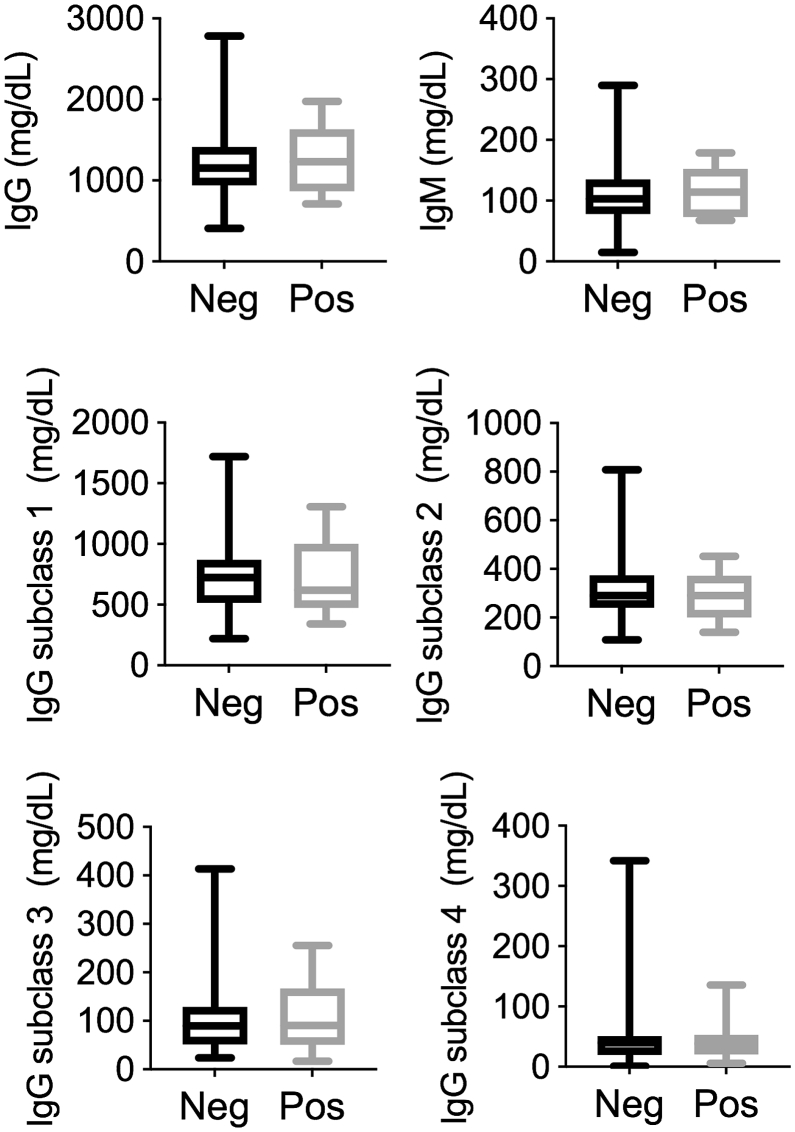
**The Presence of Autoantibodies is Not Associated with Levels of Total IgG, IgG subclasses or IgM.** Levels of total antibody classes (IgG and IgM) and subclasses (IgG1, IgG2, IgG3, IgG4) were measured for autoantibody negative (Neg, n ​= ​43) versus positive (Pos, n ​= ​14) specimens from patients without history of autoimmune disorders. Box-and-whisker plots show median and 25th to 75th percentiles; whiskers indicate minimum and maximum values. No antibody levels were found to be statistically different between groups using the Mann–Whitney U test.

**Fig. 4:**
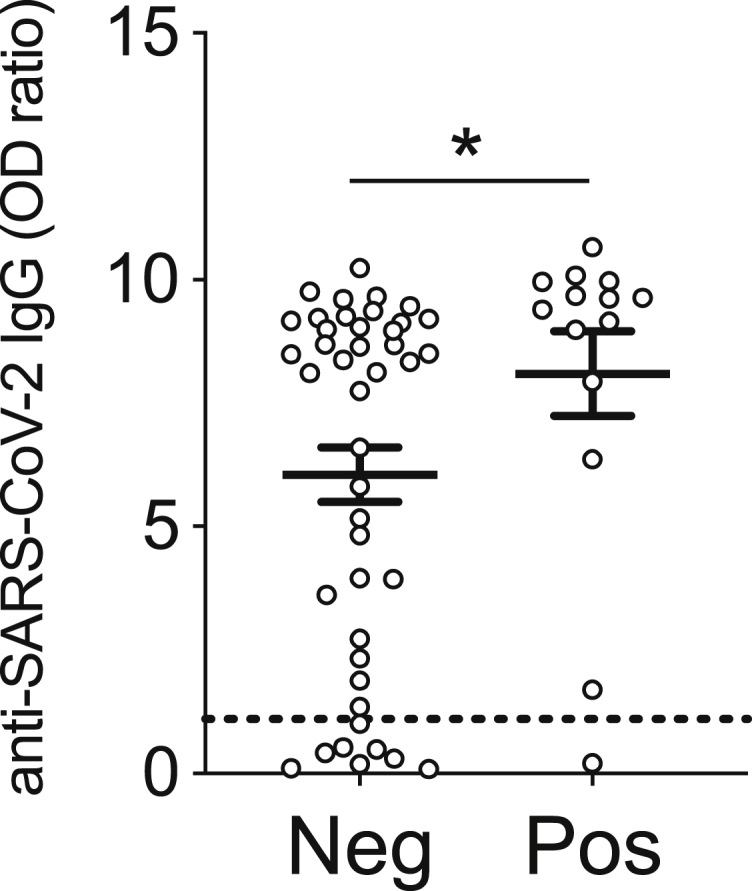
**Increased anti-SARS-CoV-2 Antibodies in Autoantibody Positive Patients.** Levels of anti-SARS-CoV-2 IgG were measured for autoantibody negative (Neg, n ​= ​43) versus positive (Pos, n ​= ​14) specimens. The dotted line indicates the manufacturer’s suggested threshold for positivity. Mean±SEM is shown. ∗*p ​=* 0.0086 using the Mann–Whitney U test.

We sought to determine whether the presence of autoantibodies correlated with laboratory values indicating inflammation. For this analysis, we excluded patients with pre-existing history of immunologically-mediated disorders to minimize the confounding effect of pre-existing autoimmunity-related factors. We found no difference in WBC, C-reactive protein (CRP), ferritin, fibrinogen or IL-6 between patients who were autoantibody positive or negative (Fig. 2). We then examined levels of total IgG, IgG subclasses and IgM and found no difference in any antibody class or subclass between patients who were autoantibody positive or negative (Fig. 3). Finally, we measured anti-SARS-CoV-2 spike protein (S1 domain) IgG antibodies and found increased antibody levels in autoantibody positive versus negative specimens (Fig. 4). The majority of patients in both groups were positive for anti-SARS-CoV-2 antibodies (83.7% of autoantibody negative vs 92.9% of autoantibody positive, *p* ​= ​0.66).

## 4 Discussion

Exuberant immune responses are a hallmark of COVID-19, but the mechanisms leading to immune-mediated damage are unclear. In the present study, we evaluated antibodies to nuclear and other autoantigens in specimens from hospitalized patients with acute COVID-19. Overall, we found that 30% of patients had detectable antibodies to nuclear or phospholipid antigens. Among patients without pre-existing immunologically-mediated disorders, the overall frequency of autoantibodies was 26%. Autoantibody positive patients were more likely to have severe disease requiring care in the ICU and higher levels of anti-SARS-CoV-2 antibodies, but did not have elevated inflammatory markers or total immunoglobulin levels compared to autoantibody negative patients. Longer term follow-up could reveal whether inflammatory markers or levels of immunoglobulin classes or IgG subclasses are altered after COVID-19 and confer susceptibility to chronic infection or inflammation.

Of patients with any immunoreactivity, 84% had antibodies to nuclear antigens. Recent data suggest a high prevalence of antinuclear antibodies in severe acute COVID-19 [10,11]. Our findings were generally similar, as 42% of patients in our cohort who received ICU care had detectable antibodies to nuclear antigens. Antinuclear antibodies are a hallmark of several autoimmune diseases [9], but also commonly present in acute illness, including infections [22,23]. The frequency of antinuclear antibody detection in healthy individuals depends on the methodology and threshold for positivity [24]. Reference range studies using specimens from healthy controls can guide individual clinical laboratories in setting thresholds to provide clinically meaningful results [25]. We found that 12% of normal donors in our region had a positive antinuclear antibody test using the manufacturer’s suggested thresholds for our assay, but the quantitative values for these healthy controls were only slightly above the threshold for positivity. Thus, we previously established laboratory-defined cutoffs with an indeterminate range to encompass these weakly-reactive healthy controls. The majority of acute COVID-19 patients (14 of 16, 88%) with a detectable ANA were in this weakly-reactive, indeterminate classification, and the two remaining patients with a strongly positive antinuclear antibodies had a known prior history of systemic lupus erythematosus and autoantibodies. Thus, in our cohort we did not find any patients who developed strongly reactive antinuclear antibodies in response to acute SARS-CoV-2 infection.

Other acute infections have been associated with detectable ANA that were not indicative of subsequent autoimmune disease, but rather reflected transient autoreactive B and plasma cell activation [22]. One limitation of our study is that we focused on patients in the acute phase of infection and do not yet know whether the detectable antibodies to nuclear antigens will persist with disease resolution and viral clearance, or whether additional autoreactivity will develop later in the course of disease or during convalescence. Recent evidence suggests that severe COVID-19 is associated with acute extrafollicular B cell expansion, including clonotypes previously associated with autoreactivity [26], although the long-term persistence of these clones is not yet known. Thus, our findings could reflect either transient immune activation in the setting of acute infection, or early loss of tolerance predicting a path toward chronic autoimmunity. Future studies will be needed to determine the long-term consequences of SARS-CoV-2 infection. These studies could determine whether the low-level autoantibody responses we described in the acute phase of infection increase or decrease over time. In addition, careful clinical follow-up could include observation for signs of chronic autoimmune disease.

Although antiphospholipid syndrome is an emerging concern in COVID-19, we did not find evidence of a high prevalence of antiphospholipid antibodies in our cohort, as only 9% of patients were positive for any antiphospholipid antibody. These results are in agreement with a study from Spain, which focused specifically on COVID-19 patients with venous thromboembolism, and also found a low prevalence of antiphospholipid antibodies [27]. Similar to antinuclear antibodies, antiphospholipid antibodies can be detected transiently in patients without autoimmunity, particularly in the setting of acute illness and infection [28,29]. These transient antiphospholipid antibodies are not clearly pathogenic, and diagnosis of antiphospholipid syndrome requires antibody detection over time. Future studies with long-term follow up will be needed to determine whether antiphospholipid antibodies are involved in thromboembolism during acute SARS-CoV-2 infection and whether they persist in convalescent patients after recovery.

## Credit author statement

L. Angelica Lerma: Data curation, Formal analysis, Writing – original draft, Writing – review & editing, Anu Chaudhary: Investigation, Data curation, Writing – review & editing, Andrew Bryan: Resources, Writing – review & editing. Chihiro Morishima: Resources, Writing – review & editing. Mark H. Wener Conceptualization, Resources, Writing – review & editing, Susan L. Fink Conceptualization, Data curation, Resources, Formal analysis, Writing – original draft, Writing – review & editing.

## Declaration of competing interest

The authors declare that they have no known competing financial interests or personal relationships that could have appeared to influence the work reported in this paper.
